# Phylogenetic tree-based amino acid sequence generation for proteomics data analysis of unknown species

**DOI:** 10.1016/j.csbj.2025.05.041

**Published:** 2025-05-29

**Authors:** Nobuaki Miura, Tsuyoshi Tabata, Yasushi Ishihama, Shujiro Okuda

**Affiliations:** a Division of Bioinformatics, Niigata University Graduate School of Medical and Dental Sciences, 2-5274 Gakkocho-dori, Chuo-ku, Niigata 951-8514, Japan; b Graduate School of Pharmaceutical Sciences, Kyoto University, Kyoto 606-8501, Japan; c Medical AI Center, Niigata University School of Medicine, 2-5274 Gakkocho-dori, Chuo-ku, Niigata 951-8514, Japan

**Keywords:** Amino acid sequence generation, Proteomics data analysis, Peptide identification, Spectral matching, Random branch, Ion Cover Score

## Abstract

In bottom-up proteomics, selecting an appropriate protein amino acid sequence database is vital for reliable peptide identification. However, this approach excludes species with unsequenced genomes, limiting the comprehensiveness. This is a major challenge in current microbiota proteomics, a rapidly developing field, which involves simultaneously assigning proteins to species in a sample and analyzing them using databases of protein amino acid sequences with known genomes. We aimed to develop a method to extend the database species diversity by generating protein amino acid sequences of unknown species using phylogenetic relationships among known species. To evaluate this approach, we generated the *Helicobacter pylori* F16 strain sequence based on the phylogenetic relationships of 29 closely related strains (excluding F16). Consequently, the percentages of peptides that matched the peptides obtained from the reference F16 strain increased by 5 %, based on sequence generation. Proteomics data analyses were performed on the F16 strain using the generated sequence database to validate peptide identification. Peptide spectral match decreased when the database was expanded using sequence generation owing to a decrease in sensitivity primarily caused by an increase in decoy hits. The decrease in identification sensitivity caused by large-scale databases could be improved by introducing a novel score, Ion Cover Score, based on spectral matching. The sequence generation method used in the present study and the introduction of scores based on spectral matching could accelerate proteomics development.

## Graphical Abstract

**Figure:**
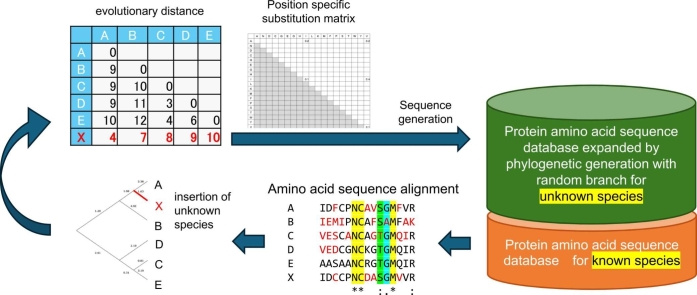

## 1 Introduction

The quality of mass spectrometry (MS)-based proteomics is fundamentally determined using database construction. Genomic data is essential for proteomics involving unknown species, particularly when numerous unidentified species are present. The recommended approach is to perform metagenomic analysis on the sample and construct a protein amino acid sequence database based on the metagenome. Metagenomes represent the collective genetic material within a specific environment, encompassing all microorganisms such as bacteria, viruses, and fungi.

An example of applying proteomics to unknown species is investigating gut microbiota, which comprises diverse microorganisms, including bacteria, viruses, fungi, and other microbes, in the digestive tracts of humans and animals. These microorganisms play essential roles in digestion, nutrient absorption, and the immune system. The roles of gut microbiota in maintaining homeostasis and the mechanisms underlying gut microbiome abnormalities associated with disease, which could not be determined through only the human genome, are currently being elucidated using proteomics [1], [2], [3]. In contrast to genomic and metagenomic analyses that provide static data, advanced proteomics offers dynamic information on expressed proteins and species, offering insights into conditions such as obesity [4], diabetes [5], inflammatory bowel disease [6], [7], colorectal cancer [8], human immunodeficiency virus [9], [10], acute leukemia [11], and autoimmune liver diseases [12]. Metagenomic analysis is used to investigate microbial community composition and function [13]. However, the quality of amino acid sequences derived from metagenomes depends on metagenomic data accuracy. Additionally, metagenomic sequencing is expensive, making it inaccessible to all researchers. In many cases, publicly available databases generated from previous analyses, such as those from the National Center for Biotechnology Information [14], UniProt [15], the Human Microbiome Project, and Metagenomics of the Human Intestinal Tract [16], [17], [18], [19], and other resources such as the Human Microbiome Project [20], Culturable Genome Reference [21], Human Gastrointestinal Bacteria Culture Collection [22], and the Unified Human Gastrointestinal Protein catalog [23], are used.

Owing to high species diversity in organisms, even the most extensive available database may lack protein sequences. In addition, using large databases can reduce peptide identification sensitivity and increase false identifications owing to the limitations of conventional target-decoy (TD) searches [1], [24], [25], [26]. TD searching is used to estimate the false discovery rate (FDR) of peptide identifications by comparing search results against a database containing real (target) and decoy (false) sequences. To mitigate challenges regarding false discovery, several methods have been developed, including two-step [27], multi-step [28], sectioning [29], percolator [30], and tool-independent and data-dependent peptide spectral match (PSM) rescoring methods (TIDD) [31]. Additionally, alternative FDR evaluation approaches, such as direct FDR calculation [32], the Benjamini–Hochberg method [33], and *de novo* analysis [25], [34], [35], [36], have been proposed. Similarly, spectral matching-based scoring methods, such as AlphaPept’s generic score and Morpheus [37], [38] are currently being investigated. However, because these methods fundamentally rely on the TD approach, they remain subject to score distributions of target and decoy PSMs. Score distribution changes with database expansion, and previously identified peptides may not be identified with the expanded database. That is, the additive nature of the method would not be satisfied. In large-scale databases, the likelihood of excluding true peptides exceeds the false positive rate guaranteed by FDR. Therefore, absolute standards for peptide identification are necessary when using large-scale databases.

In the present study, we proposed a random branching method to generate sequences for unknown species based on phylogenetic relationships among known species, facilitating unknown species identification in proteomics. To validate this procedure, we conducted proteome analysis of the *Helicobacter pylori* F16 strain using MS data and a protein amino acid sequence database generated using the random branching method. Furthermore, we developed a method to mitigate sensitivity loss in proteomics caused by large-scale database usage. We introduce a novel score, Ion Cover Score (ICS), from a tandem mass spectrometry (MS/MS) spectral matching perspective, and discuss the decrease in sensitivity and false positive rates when using extensive databases such as that used in the present study.

## 2 Material and methods

### 2.1 Generation of amino acid sequence using the phylogenetic information of known species

**Fig. 1:**
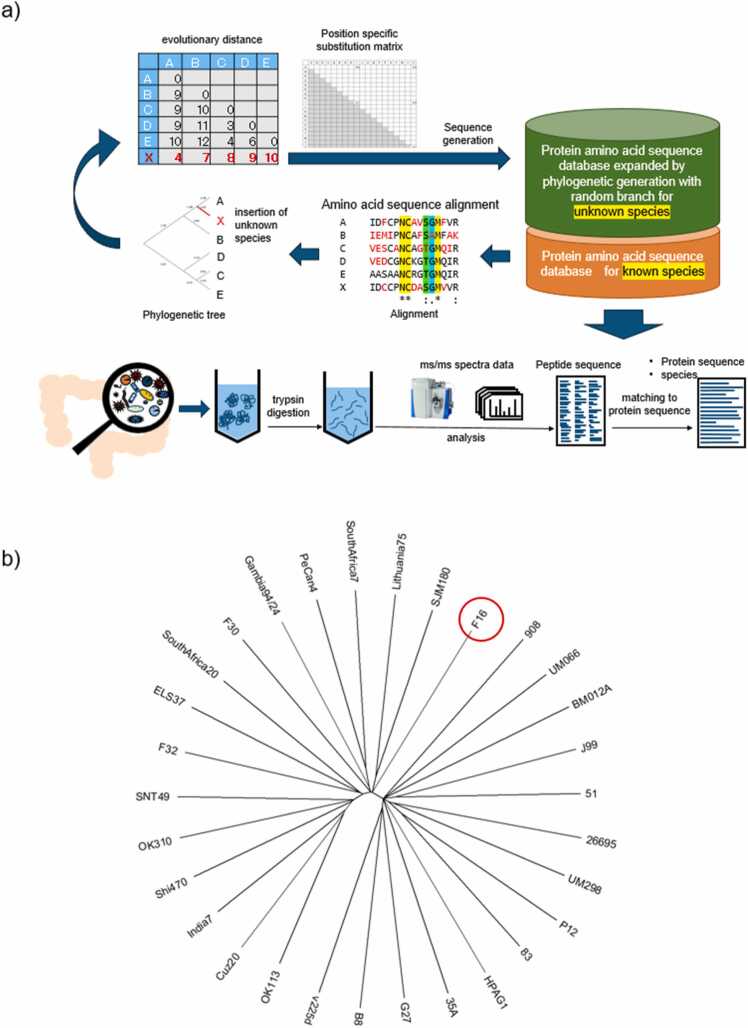
Overview of sequence generation and evaluation. a) Workflow of the strategy for protein sequence generation and data analysis with the expanded database. b) Phylogenetic tree of F16 and pylori29 used in the present study. This was created using MEGAX [39] based on the maximum likelihood method with evolutionary distance as the metric. The "Standard" option of MEGAX was used for the conversion from genome to amino acid.

Fig. 1 presents a schematic of the sequence generation process we developed. A database was constructed by incorporating extended protein amino acid sequences generated based on the phylogenetic relationships of known species, and MS data from fecal samples were analyzed. The extended amino acid sequence database, highlighted in green in Fig. 1, was used for peptide identification, protein estimation, and taxonomy estimation.

#### 2.1.1 Protein amino acid sequences and raw MS data used in this study

The amino acid sequences of proteins for the *H. pylori* F16 strain (F16) were extracted from GenBank entry AP011940.1 using our in-house program. Additionally, the amino acid sequences of 29 strains closely associated with the F16 strain (pylori29) were obtained using the GenBank entries listed in Table S1. A phylogenetic tree for pylori29 and the F16 strain is shown in Fig. 1b.

The MS raw data obtained from the proteomic analysis of the *H. pylori* F16 strain, conducted by Sugiyama et al. [40] were downloaded from the ProteomeXchange Consortium (http://proteomecentral.proteomexchange.org) via the jPOST partner repository (https://jpostdb.org) [41] under the dataset identifier PXD011364.

#### 2.1.2 Phylogenetic classification with the protein amino acid sequence database of known species

**Fig. 2:**
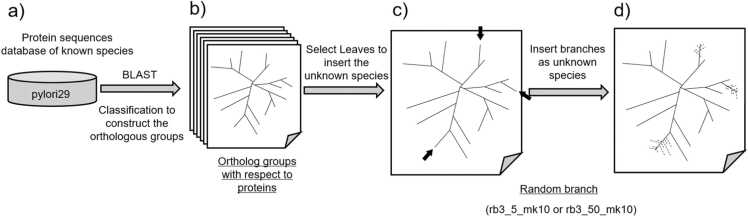
Conceptual overview of the random branch method for constructing orthologs from a known species sequence database and inserting branches for unknown organisms. a) Database for proteins closely related to *Helicobacter pylori* F16 strain (pylori29), used as the database and query sequences for BLAST searches to construct orthologous groups. b) Orthologous group construction using BLAST results. Homologous groups were formed, and the evolutionary distances between each leaf were calculated. The phylogenetic tree represents this process, depicting multiple orthologous groups within a dataset. c) Random selection of leaves from the phylogenetic tree. d) Random insertion of branches representing unknown species (X) at a defined phylogenetic distance from selected leaves.

The phylogenetic relationships of pylori 29 were investigated, and sequences of unknown strains were generated as follows. To construct orthologous groups, we calculated the sequence identity among all pylori29 sequences through a BLAST search using the amino acid sequence (sequence) of all Pylori29 proteins as the database, with all pylori29 sequences as queries [42]. BLAST results for all query sequences were clustered using the K-means method [43] based on BLAST score and identity per total sequence to extract the most closely related clusters of query sequences and establish links between compatible query and result sequences. In this process, the BLAST search was performed using all sequences as queries, generating two-way matching. By linking the constructed sequences, all proteins were categorized into orthologous groups (Fig. 2b). MAFFT alignments of amino acid sequences were performed for each orthologous group to determine evolutionary distances (number of substitutions) between them [44]. The ortholog group construction was performed using our in-house program, with BLAST results as input. Based on this workflow, Fig. 2b illustrates a diagram featuring a phylogenetic tree icon representing the orthologous group.

#### 2.1.3 Random branch (rb) for insertion of unknown species

We assumed a phylogenetic relationship among sequences within the constructed ortholog group and considered the included sequences as leaves. For instance, we randomly selected three protein sequences corresponding to the leaves of each group (Fig. 2c) and inserted 5 or 50 branches, representing an unknown strain X, at randomly determined evolutionary distances between these leaves and their nearest neighbors (Fig. 2d). This was termed the rb method. To describe the rb type, we used "rb," the number of selected leaves L (n = 3), and the maximum number of inserted branches B (5 or 50), denoted as rbL_B (e.g., rb3_5 or rb3_50). The sequence of the unknown species X was stochastically generated using the sequence of the most closely related species as the initial sequence, followed by repeated substitutions based on random numbers derived from the position-specific substitution matrix (PSSM) within the group. In probabilistic generation, where random numbers were used, an rb was further denoted by appending "mkN" when maximum N sequences were generated per branch. For instance, a maximum of 10 sequences generated per branch for rb3_5 and rb3_50 were denoted as rb3_5_mk10 and rb3_50_mk10, respectively.

#### 2.1.4 Stochastic generation of the unknown species sequences

**Fig. 3:**
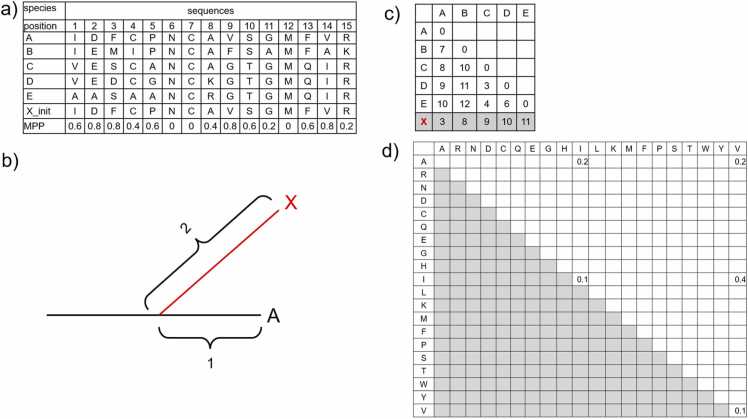
Specific method for generating protein amino acid sequences based on the random branch method. a) Amino acid sequences of orthologous groups A–E from known organisms, the selected initial sequence X_init, and mutation probability with respect to the position (MPP), which represents the number of substitutions from the initial sequence in A–E. b) A new branch was added when the unknown species X was inserted, originating from A with a branch length of 2, yielding a total length of 3 from leaf A. c) Evolutionary distances between known species A–E and the evolutionary distance from X to A–E. d) Position-specific substitution matrix (PSSM) at the first position of the table in Fig. 3a.

The sequence of the unknown species X, inserted using the rb method, was generated as follows, with organisms A–E serving as known species references (Fig. 3a). We assumed that X was inserted near Leaf A in the phylogenetic tree shown in Fig. 3b. The evolutionary distance (number of substitutions) from X to the closely related species A–E was calculated and added to the distance matrix from A to E (Fig. 3c). Sequence generation for X was based on repeated substitutions, constrained by an evolutionary distance condition that will be described later. Evolutionary distances were determined by aligning X with A-E for each substitution iteration. If the final distance condition, as shown in Fig. 3c, was met, the sequence was accepted as a valid generated sequence.

The substitution frequencies for each position in the closely related species (Fig. 3a) were calculated for the initial X sequence, quantifying the likelihood of amino acid substitutions. Similarly, the PSSM for the closely related species (Fig. 3d) was computed to determine the probability of amino acid substitution at specific positions. For example, at the first position in Fig. 3a, there were four substitutions between I and V; I remained I, V remained V, and two substitutions occurred each between I and A and V and A. The PSSM represents these frequencies converted into probabilities. Because a limited number of substitutions can be obtained from the PSSM derived from pylori29, we incorporated additional probabilities from the general substitution matrix BLOSUM95, scaled by a factor of 0.01. Substitutions continued using these probabilities until the evolutionary distance condition was met and the specified N sequences in mkN were generated, or the maximum substitution threshold (10,000) was reached.

We considered the following three evolutionary distance conditions:

1. “strict,” generated sequence satisfies all the substitution distances shown in Fig. 3c.
2. “nearest,” generated sequence is accepted if it is farther than the distance to the nearest neighbor in evolutionary distance, i.e., if the evolutionary distance is ˃ 3 for all of B-E in the present case in Fig. 3b.
3. “order,” generated sequences are accepted if the order of evolutionary distance to each ortholog is preserved.

In all cases, the evolutionary distance condition for the nearest neighbor, A, was set at 3.

### 2.2 MS data analysis

For MS data analysis, we constructed a sequence database by performing trypsin digestion of the protein sequence of interest, allowing zero missed cleavages. Duplicate sequences were removed. Additionally, I and L were treated as identical amino acids when comparing sequences.

We identified peptides through the constructed peptide database using MaxQuant v2.5.2.0 (MQ) [45] and Comet 2019.01 [46]. MQ was executed with default parameters using an undigested protein amino acid sequence database. The FDR cutoff was set to 0.01. For Comet analysis, an *in silico* digested peptide sequence database was used; however, no further *in silico* digestion was performed within Comet, and only tryptic peptides were included in the search. The option for random fragmentation of peptide sequences was disabled. The minimum peptide length was set to seven amino acid residues. Comet search was conducted by selecting the best Xcorr peptide among five candidate peptides per spectrum and assigning one peptide per spectrum. The decoy database was generated by reversing each sequence in the digested peptide database, except for the C-terminal residue (typically lysine or arginine). The parameter files used for the MQ and Comet calculations are included as Dataset S1 and S2, respectively, in the Supporting information.

### 2.3 ICS for evaluating the quality of MS/MS spectral matches

**Fig. 4:**
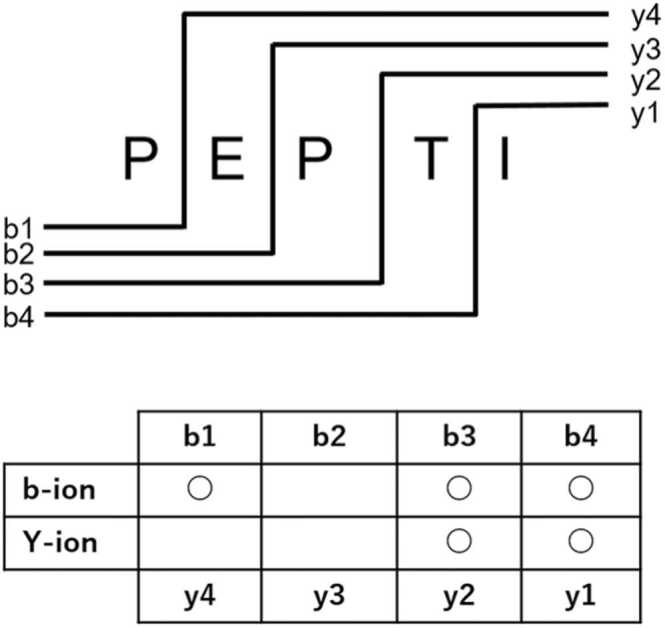
Ion Cover Score (ICS) score concept. The ICS is calculated as a percentage based on the number of detectable ions derived from the sequence length and the number of detected b- and y-ions.

To assess the reliability of the PSMs obtained from proteomic analysis, we defined the ICS score, which is calculated as the ratio of predicted b- and y-ions from *in silico* fragmentation of the parent ion in MS/MS to experimental b- and y-ions (Fig. 4):

$$\mathrm{ICS}=\frac{N_{b-\mathrm{ion}}+N_{y-\mathrm{ion}}}{2(L_{\mathrm{sequence}}-1)},$$

where *N*_b-ions_ and *N*_y-ions_ are the numbers of b-ions and y-ions detected, respectively, and *L*_sequence_ is the sequence length. Because ICS increases with the number of matching ion spectra, an ICS closer to 1 suggests a higher reliability of peptide identification. Using the XLM output file from Comet and the MGF file generated from the raw mass spectrometry data, we calculated the masses of y- and b-ions produced through the fragmentation of precursor peptide sequences. *N*_y-ion_ and *N*_b-ion_ values were obtained by matching these masses to the MS/MS peak masses in the mgf file within a 20 ppm range.

### 2.4 Peptide identification through TD search and rescoring using Percolator

Using the results of the Comet database search, we conducted a traditional TD search to identify peptides. The peptide sets obtained from the Comet search were sorted using Xcorr scores, and those with a 2D/(T + D) value < 0.01 were accepted (Comet); where T and D are the numbers of target and decoy PSMs, respectively, and are ranked from highest to lowest score. Additionally, PSMs were rescored using Percolator 3.5.0 [30] (referred to as "Comet/PCL"). In Percolator, PSMs were evaluated using Comet results and parameters such as RT, ExpMass, CalcMass, lnrSp, deltLCn, deltCn, lnExpect, Xcorr, Sp, IonFrac, Mass, PepLen, Charge1, Charge2, Charge3, Charge4, Charge5, Charge6, enzN, enzC, enzInt, lnNumSP, dM, and absdM. The q-value cutoff was set to 0.01.

Percolator was executed with ICS, incorporating the number of matches (*N*_y-ion_+*N*_b-ion_) and peptide length as parameters in Comet/PCL to refine spectral matches in the scoring process (Comet/PCL(ICS)). The q-value cutoff remained at 0.01.

The number of proteins was counted using the simplest method: searching a protein amino acid sequence database to determine the number of protein sequences containing peptides identified in MS-based proteomic analysis.

## 3 Results and discussion

### 3.1 Reproducibility of generated protein sequences

Table S2 presents the results obtained from generating a maximum of 10 sequences per branch for rb3_50 (rb3_50_mk10) using the three generation methods, namely strict, order, and nearest. Because PSMs identified during MS analysis will be discussed later, the results are shown based on the number of peptides. Protein amino acid sequences underwent trypsin digestion *in silico*, allowing for no missed cleavages. The number of peptides digested from the generated sequences were 1618,632, 2713,423, and 2663,548 for strict, order, and nearest, respectively. Fewer peptides were generated in strict than in order and nearest, with the numbers for order and nearest being nearly identical. This indicates that "strict" involves the application of the most rigorous conditions for sequence generation. The percentages of peptides matching those from the reference F16 sequence (reproduction percentage) were 94.3 %, 94.8 %, and 94.8 % for strict, order, and nearest, respectively. The reproduction percentage for the F16 sequence was similar for order and nearest. Regarding the reproduction percentage of the F16 strain, order and nearest were nearly identical and yielded a high match. In principle, strict is the most appropriate phylogenetic method; however, given the need to generate more sequences while considering computational constraints, we will use the simpler nearest condition with a higher reproduction percentage for the subsequent discussion.

**Fig. 5:**
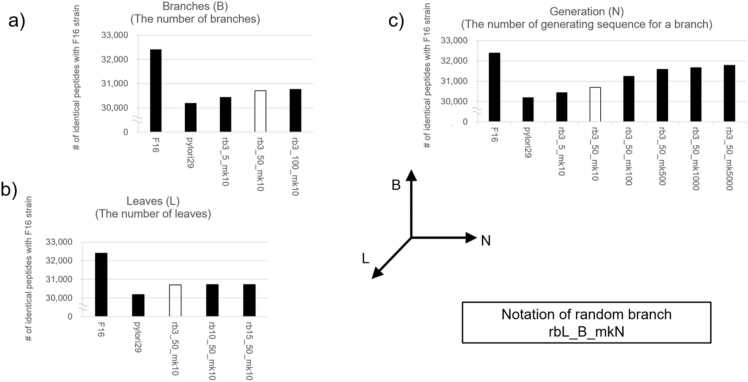
Effect of random branch method parameters. Bar charts showing the number of peptides identical to those in F16 when the parameters are varied: (a) number of branches (B), (b) number of leaves (L), and (c) number of peptide generations (N). White bars indicate results under the same parameter, rb3_50_mk10. Values below 29,000 on the y-axis have been truncated.

Fig. 5 shows the number of peptides digested from generated sequences in the rb method that correctly matched the digested peptide sequence of the F16 strain when varying the number of leaves (L), the maximum number of branches inserted per leaf (B), and the number of sequences generated per branch (N) as parameters. The number of non-redundant *in silico* tryptic-digested peptides obtained from the F16 sequence was 32,398. Among the 161,550 non-redundant tryptic-digested peptides of Pylori29, 30,185 were identical to those in F16, yielding a reproduction percentage of 93.2 %, indicating that these peptides were detectable without sequence generation. Increasing the number of branches (B-axis in Fig. 5) slightly increased the reproduction percentage, whereas the number of leaves (L-axis) had no significant impact. Because leaves were randomly selected in each orthologous group in the rb method, their overall contribution remained homogenized. This suggests that a relatively small number of leaves may be sufficient. The most significant effect was observed in the maximum number of peptides produced per branch (N-axis), indicating that sequence diversity plays a crucial role.

In rb5_50_mk100, which generated approximately 100 sequences per branch, 31,246 peptides matched those of F16 (96.4 % reproduction percentage). In rb3_50_mk5000, 31,778 peptides matched, achieving a 98.1 % reproduction percentage. Compared with Pylori29, which only collected closely related species, rb3_50_mk5000 increased the reproduction percentage by approximately 5 %. This suggests that the rb method is effective for predicting sequences of unknown organisms. The statistics for each parameter are presented in Table S3. Although the random branching can sufficiently cover sequences of unknown species, it requires generating numerous sequences owing to sequence randomness. The rb3_50_mk5000, with a reproduction percentage exceeding 98 %, generated 650 million sequences and 83 million non-redundant peptides. As an example of the computational time required for the rb method, generating rb3_50_mk100 required approximately 100 h using six nodes (216 cores) on a server equipped with Xeon(R) Gold 6154 @ 3.00 GHz (36 cores). For the following calculations, we used rb3_5_mk10 and rb3_50_mk10 from a computing resources perspective.

### 3.2 Validation using F16 MS data

**Fig. 6:**
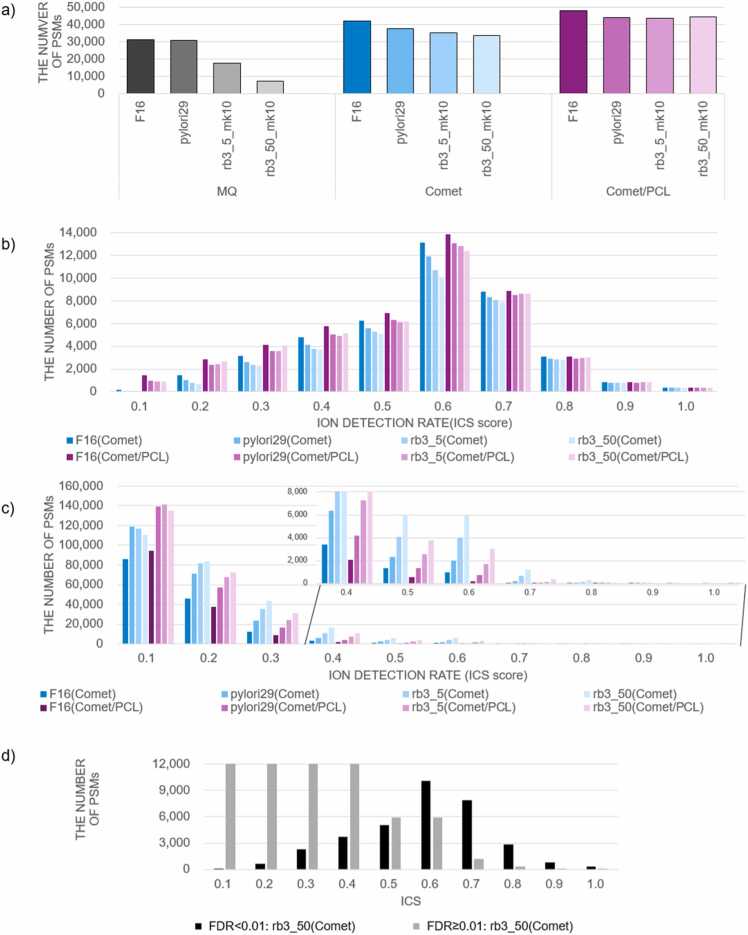
Number of peptide-spectrum matches (PSMs) obtained in the proteomic analysis of *Helicobacter pylori* and distribution of the ion cover score (ICS). a) The number of PSMs with FDR < 0.01 obtained through MQ, Comet, and Comet/PCL analyses. b) ICS distribution for PSMs with FDR < 0.01 obtained through Comet and Comet/PCL analyses. The ICS on the horizontal axis is less than the indicated value and is equal to or greater than the value of the left neighbor. For example, an ICS of 0.6 indicates that 0.5 ≤ ICS < 0.6. c) ICS distribution for PSMs with FDR < 0.01 obtained through Comet and Comet/PCL analyses. The zoomed-in view for ICS ≥ 0.4 is shown on the upper right side. d) ICS distribution for PSMs obtained with FDR < 0.01 and PSMs rejected with FDR ≥ 0.01 for the rb3_50_mk10 database in the Comet analysis.

Fig. 6a shows the number of PSMs obtained through three methods: MQ, Comet, and Comet/PCL. When a large database is used, the number of PSMs does not necessarily increase with database expansion for conventional methods such as MQ and Comet. Using the rb3_50_mk10 database, the numbers of PSMs detected were 7125 and 33,727 for MQ and Comet, respectively. These detections accounted for 23 % and 80 % of the PSMs based on the F16 strain database, despite 94.8 % of the generated peptide sequences being present in the F16 strain, as mentioned in Section 3.1. The MQ results indicate that the PSM count significantly decreased when the database is extended via the rb method compared with Comet. This is related to the shift in score distribution for randomly matched decoy PSMs, causing an upward movement in the score threshold as the database expands (Fig. S1). However, for Comet/PCL, the number of PSMs increased slightly with database expansion as the sequence count increased.

### 3.3 Validation for MS data using ICS

The reduced sensitivity in large sequence databases during conventional TD searches depends on the relative distribution of target and decoy PSMs. The ICS distribution was calculated to evaluate the spectral match between PSMs obtained with FDR< 0.01 (Fig. 6b) and those rejected with FDR ≥ 0.01 (Fig. 6c). The ICS distribution for PSMs obtained using Comet and Comet/PCL with FDR < 0.01 is shown in Fig. 6b. Notably, approximately 5000–7000 PSMs (over 15–20 % of all PSMs) with ICS < 0.3 were found in Comet and Comet/PCL (FDR < 0.01). In MS/MS spectrum identification, ICS refers to the ratio of the number of ions actually detected to the number of ions expected based on the precursor sequence length. That is, ICS is used to measure spectral matching quality. Therefore, when ICS is low (e.g., <0.3), the corresponding PSM may be undesirable. This effect was more pronounced when using a percolator, as Comet/PCL generated more PSMs than Comet alone and also increased the number of PSMs with ICS < 0.3. Figure S2 shows the cumulative sum of PSMs for each ICS value as a percentage of the total PSMs. For Comet and Comet/PCL, the distributions remained nearly identical regardless of database size. In the ICS < 0.5 region, the Comet/PCL percentage was slightly higher. This is because certain PSMs with low ICS values persisted among PSMs with FDR < 0.01, potentially owing to the nature of Xcorr scoring and rescoring in Comet/PCL. The ICS distribution for PSMs discarded with FDR ≥ 0.01 is shown in Fig. 6c, indicating that many discarded PSMs had high ICS values (e.g., ICS ≥0.5). For instance, approximately 6000 and 3000 PSMs were found in Comet and Comet/PCL, respectively, within the 0.5 ≤ ICS < 0.6 range when the rb3_50_mk10 database was used. This suggests that conventional methods cause the rejection of many PSMs with high ICS and reliability. Fig. 6d illustrates the ICS distribution for PSMs with FDR < 0.01 (black) and FDR ≥ 0.01 (gray) from the Comet analysis using the rb3_50_mk10 database. Because FDR is determined through the distribution of target and decoy PSMs, these findings suggest the potential for directly assessing PSM reliability using ICS as an indicator.

PSMs with ICS ≥ 0.4 were collected from the target database of the Comet search in rb3_50_mk10. These PSMs were compared with those obtained from Comet, and overlaps were counted (Table 1). The ICS portion increased as the database expanded, indicating that ICS retrieves PSMs rejected by Comet at FDR ≥ 0.01 as the database grows.

**Table 1:** Overlaps between peptide-spectrum matches (PSMs) obtained through Comet and those obtained directly using ion cover score (ICS) with a threshold of 0.4.

	Comet/PCL only	Co-identified	ICS≥ 0.4 only
F16	13,027	33,481	1101
pylori29	12,099	31,869	1836
rb3_5_mk10	11,991	31,510	3836
rb3_50_mk10	13,045	31,227	6384

In this study, we verified the usefulness of the random branch method by reproducing the sequences of F16 strain using closely related Pylori29. We attempted to regenerate the F16 strain by using a sequence database consisting of species that are more evolutionarily distant rather than closely related species to generate random branches. As a model set to evaluate such evolutionarily distant cases, we extended SIHUMIx [47], a set of sequences of eight representative human intestinal bacteria, with a random branch (rb8_10_mk10). Since SIHUMIx is distantly related to F16, the number of peptides matching F16 peptides is only 5.8 %. The random branch-based sequence generation increased that percentage to 14.2 % (Table S4). This suggests that random branching may be applicable to distantly related organisms across species.

Because the current rb method is based on the insertion of branches near the leaves phylogenetically, only phylogenetically close sequences (based on core genomic features) are generated. Therefore, the efficiency of generating reference-matched sequences is lower than the total number of sequences generated. To generate sequences with pan-genomic features beyond this, branches should be inserted upstream of the leaves rather than near them. Furthermore, it is necessary to expand various array generation by allowing further branches to be inserted into the previously inserted branches in a staged manner.

### 3.4 PSM reliability considerations

The number of PSMs obtained through Comet, Percolator, and ICS (with ICS ≥0.4) and the number of PSMs that matched the F16 sequence (reference-matched PSMs) are presented in Table 2. Following database expansion, Comet showed a decrease in total PSMs and reference-matched PSMs. In Comet/PCL, the total number of PSMs increased with database expansion, while the number of reference-matched PSMs decreased. In ICS, the number of PSMs increased, while the number of reference-matched PSMs remained almost constant. These results suggest that the number of reference-matched PSMs does not decrease with database size when PSMs are obtained using ICS. Using scores reflecting spectral matches, such as ICS, more PSMs and reference-matched PSMs could be obtained than with common methods such as Comet/PCL.

**Table 2:** Peptide-spectrum matches (PSMs), reference-matched PSMs, reference-unmatched PSMs, and the ratio of reference-unmatched PSMs to PSMs.

	PSMs	Reference-matched PSMs	Reference-unmatched PSMs	%Reference-unmatched PSMs^a^
	Comet			
F16	42,060	41,849	211	0.5
pylori29	37,790	36,913	877	2.3
rb3_5	35,035	33,088	1947	5.6
rb3_50_mk10	33,727	30,677	3050	9.0
	Comet/PCL			
F16	48,048	47,586	462	1.0
pylori29	43,968	42,471	1497	3.4
rb3_5	43,501	40,174	3327	7.6
rb3_50_mk10	44,272	38,657	5615	12.7
	Comet/PCL(ICS)			
F16	49,406	48,928	478	1.0
pylori29	45,671	44,045	1626	3.6
rb3_5	45,533	41,841	3692	8.1
rb3_50_mk10	46,287	40,189	6098	13.2
	ICS≥ 0.4			
F16	34,475	34,475	0	0.0
pylori29	33,449	32,832	698	2.1
rb3_5	34,781	32,853	2218	6.3
rb3_50_mk10	36,574	32,841	4434	11.8

^a^ Ratio (percentage) of “number of reference-unmatched PSMs” to number of PSMs.

Comet/PCL (ICS) was run with ICS, incorporating the number of matched ions and peptide length as additional parameters when rescoring PSMs obtained from the Comet search with Percolator. The results indicate that Comet/PCL (ICS) produced the highest number of PSMs and reference-matched PSMs (Table 2). Given the arbitrariness of score thresholds in PSMs obtained using ICS as the threshold, these results suggest that using a TD strategy with ICS rescoring is feasible and optimal.

The number of PSMs obtained using traditional TD searches, such as MQ and Comet, decreased upon database expansion. The Percolator rescoring method (Comet/PCL) slightly increased PSMs; however, the relationship was not linear with the scale of sequence generation. This is possibly because decoy PSMs with random hits have a significant effect in large database cases. To improve this, we validated ICS, which directly reflects spectral matches. Many unreliable PSMs with low ICS were accepted under FDR control, while numerous reliable PSMs with high ICS were discarded under FDR control. PSMs were obtained using ICS as an indicator, suggesting that more reliable PSMs can be obtained by setting an appropriate threshold. Scoring methods such as ICS, which focus on spectral matching, are beneficial as they do not reduce the number of reference-matched PSMs compared with Comet or Comet/PCL.

However, owing to the arbitrariness in threshold selection, rescoring was performed with Comet/PCL (ICS) using Percolator, which involves considering ICS. This approach yielded the highest number of total and reference-matched PSMs (Table 2.). Furthermore, among the identification methods in the TD search, Comet/PCL (ICS) may be used to identify the highest number of proteins.

### 3.5 PSM false positive rate

False positive rates are crucial for validating the database generated using the rb method. Among the obtained PSMs, peptide sequences that do not exist in the original F16 database were defined as “reference-unmatched PSM,” or false identification PSMs, and their ratios, PSMs, and reference-matched PSMs are presented in Table 2. When the F16 database was used, we set the FDR to 1 %. This indicates that the reference-unmatched PSM rate, i.e., the false positive rate, for target-decoy-based Comet/PCL and Comet/PCL (ICS) was 1 %. For Comet, the reference-unmatched PSM rate was 0.5 % because the FDR was controlled as 2D/(T + D) × 100 < 1; where T represents the number of target PSMs and D denotes decoy PSMs. When ICS ≥ 0.4, the TD strategy is not used, and decoy PSMs are not included; thus, the reference-unmatched PSM rate is 0 for the F16 database.

The reference-unmatched PSM rate increases as the database size expands. In rb3_50_mk10, the database contains 1618,632 peptides, reproducing approximately 95 % of the F16 strain, as mentioned in Section 3.1. Of these peptides, approximately 30,000 (∼2 %) are related to the F16 strain, while 98 % can be estimated as biologically unrelated. This explains the exponential increase in the reference-unmatched PSM rate, or false identification, highlighting a critical challenge for future studies to improve identification efficiency. In Comet, the reference-unmatched PSM rate remained low and yielded a lower overall PSM count, with reference-unmatched PSMs reaching 9 % for rb3_50_mk10. Although Comet/PCL and Comet/PCL(ICS), which utilizes Percolator, increased the total number of PSMs, it also increased the false identification rate. When ICS ≥ 0.4, the reference-unmatched PSM rate is comparable to or slightly better than that observed between Comet and Comet/PCL, aligning closely with widely used Percolator results. However, owing to the remaining arbitrariness in setting the ICS threshold, future studies should explore how best to address this challenge.

### 3.6 Number of proteins

The number of proteins associated with the obtained PSMs was determined (Table 3). Additionally, the number of proteins obtained in Comet was only 5 % lower than those obtained in the others. This was expected because the PSMs obtained using this method were lower than those obtained from the other calculations. In the comparison between Comet/PCL and ICS, Comet/PCL yielded a higher number of PSMs and reference-matched PSMs; however, ICS was used to identify more proteins in regions with expanded databases. In Comet/PCL and Comet/PCL (ICS), database expansion caused a decrease in reference-matched PSMs similar to that in Comet, leading to a decline in the number of identified proteins. Conversely, in ICS, the number of proteins remained relatively stable, as the reference-matched PSM count did not fluctuate significantly. This suggests that the number of attributed proteins is closely linked to the number of reference-matched PSMs, indicating that ICS can provide stable protein identification, even when a large database is required to obtain reliable PSMs.

**Table 3:** Comparison of the number of proteins obtained by searching the amino acid sequence databases using peptide sequence match (PSMs) as the query peptide sequence.

	Comet	Comet/PCL	ICS≥ 0.4	Comet/PCL(ICS)
F16	1228	1253	1225	1267
pylori29	1200	1229	1225	1232
rb3_5_mk10	1172	1214	1225	1219
rb3_50_mk10	1152	1198	1217	1208

## 4 Conclusions

We proposed an rb method for constructing a highly diverse database using phylogenetic relationships among known species to expand proteomics databases. The results revealed that the F16 strain sequences could be generated correctly. This suggests that further development of this method would be valuable, advancing from strain-level verification to species and genus levels, enabling the generation of sequences for unknown species. Our current method is limited by its time-consuming and memory-intensive nature. To overcome this, we are currently developing software to improve generation efficiency and speed and facilitate metaproteomics applications for intestinal bacteria. Conducting proteomics using large databases has limitations; however, the rb method is expected to become a powerful database extension tool for metaproteomics and proteomics of species with unknown genomes. Future studies can enhance its applicability through integration with improved PSM acquisition methods.

## Data statement

The data used in this study, such as protein amino acid sequences for *H. Pylori* F16 and the related strains, are openly available in the NCBI database, and their IDs are described in the text and Table S1 in supporting information. The raw mass spectrometric data files are available in the ProteomeXchange Consortium via the jPOST partner repository with the data set identifier PXD011364. All other data, including the source codes, supporting the findings of this study will be made available by the corresponding author upon reasonable request.

## CRediT authorship contribution statement

**Yasushi Ishihama:** Writing – review & editing, Supervision. **Tsuyoshi Tabata:** Software. **Nobuaki Miura:** Writing – review & editing, Writing – original draft, Validation, Software, Methodology, Investigation, Conceptualization. **Shujiro Okuda:** Writing – review & editing, Validation, Supervision, Funding acquisition, Conceptualization.

## Declaration of Competing Interest

The authors have no conflicts of interest.
