# Supplementary material for: Phylogenetic tree-based amino acid sequence generation for proteomics data analysis of unknown species
Source: Comput Struct Biotechnol J. 2025 May 29;27:2313–22. doi: 10.1016/j.csbj.2025.05.041 (PMC12167054; doi:10.1016/j.csbj.2025.05.041)
Supplement: Supplementary file 1 — Supplementary material [file mmc1.docx]

**Supporting Information to**

**Phylogenetic Tree-Based Amino Acid Sequence Generation for Proteomics Data Analysis of Unknown Species**

Nobuaki Miura^a^, Tsuyoshi Tabata^b^, Yasushi Ishihama^b^, and Shujiro Okuda^a,c*^

^a^Division of Bioinformatics, Niigata University Graduate School of Medical and Dental Sciences, 2-5274 Gakkocho-dori, Chuo-ku, Niigata 951-8514, Japan

^b^Graduate School of Pharmaceutical Sciences, Kyoto University, Kyoto 606-8501, Japan

^c^Medical AI Center, Niigata University School of Medicine, 2-5274 Gakkocho-dori, Chuo-ku, Niigata 951-8514, Japan

* Corresponding author (Email: okd@med.niigata-u.ac.jp, phone: +81 25 227 0390

Table of Contents

**Figure S1.** Distribution of target and decoy peptide-spectrum matches (PSMs) for Comet search with F16 and rb3_50_mk10 databases.

**Figure S2.** Cumulative ratio of ICS distribution for identified PSMs in Comet and Comet/PCL with FDR<0.01.

**Table S1.** Twenty-nine *Helicobacter pylori* (pylori29) strains used in this study

**Table S2.** Number of peptides digested from the generated protein amino acid sequences, number of peptides similar to the original F16 sequences, and reproduction percentage ratio for F16 sequences across various random branch generations

**Table S3.** Number of proteins and non-redundant digested peptides in the amino acid sequence database for F16, pylori29, and rb3_5, rb3_50.

**Dataset S1.** Input parameters for MaxQuant calculations of raw files for the *Helicobacter* *pylori* F16 strain with the F16 database

**Dataset S2.** Input parameters for Comet calculations of raw files for the *Helicobacter* *pylori* F16 strain with the F16 database.


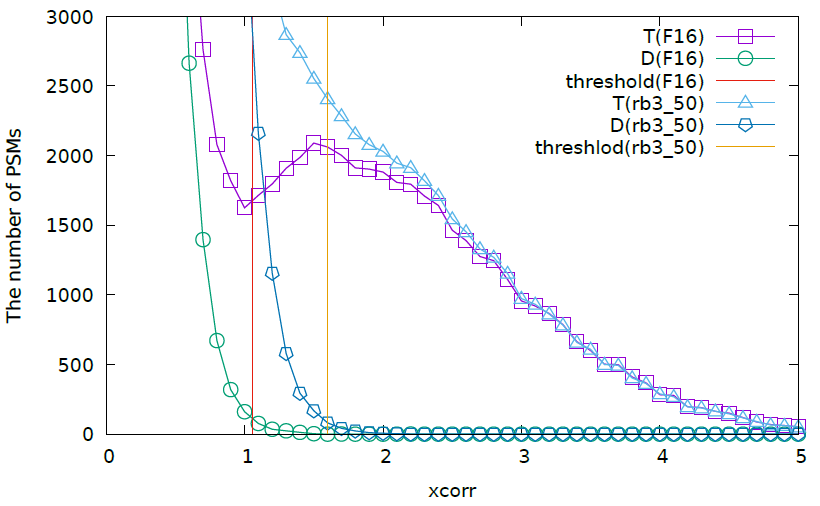


**Figure S1.** Distribution of target and decoy peptide-spectrum matches (PSMs) for Comet search with F16 and rb3_50_mk10 databases.

**
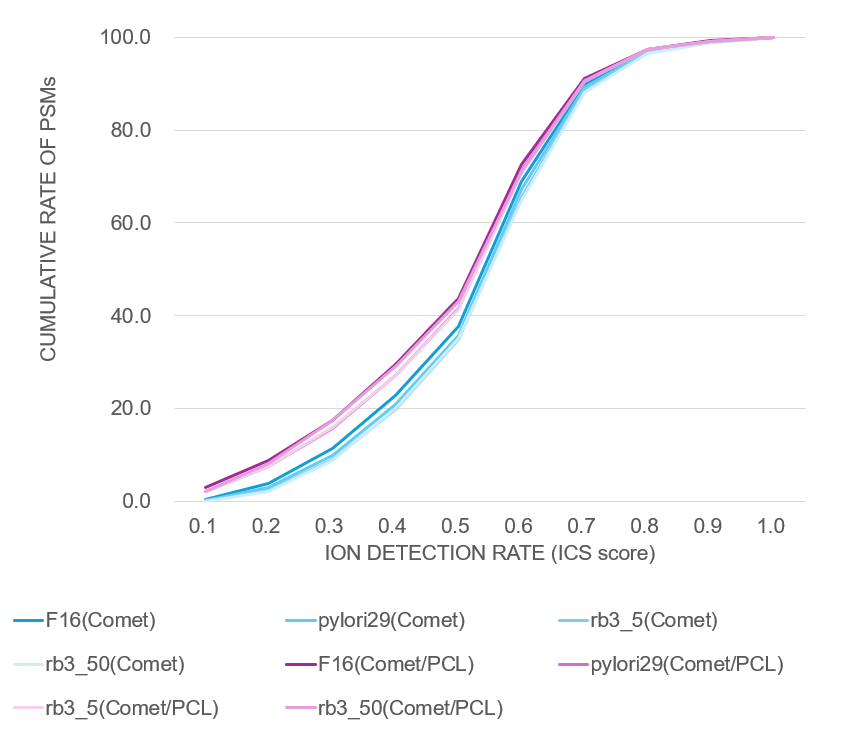
**

**Figure S2.** Cumulative ratio of ICS distribution for identified PSMs in Comet and Comet/PCL with FDR<0.01.

**Table S1.** Twenty-nine strains of *Helicobacter pylori* (pylori29) used in this study.

| *Helicobacter pylori* 26695 (NC_018939.1)^a^ | *Helicobacter pylori* F30 (AP011941.1) |
| --- | --- |
| *Helicobacter pylori* F32 (AP011943.1) | *Helicobacter pylori* G27 (CP001173.1) |
| *Helicobacter pylori* HPAG1 (CP000241.1) | *Helicobacter pylori* J99 (CP011330.1) |
| *Helicobacter pylori* OK113 (AP012600.1) | *Helicobacter pylori* OK310 (AP012601.1) |
| *Helicobacter pylori* P12 (CP001217.1) | *Helicobacter pylori* Shi470 (CP001072.2) |
| *Helicobacter pylori* 35A (CP002096.1) | *Helicobacter pylori* 51 (CP000012.1) |
| *Helicobacter pylori* 83 (CP002605.1) | *Helicobacter pylori* 908 (CP002184.1) |
| *Helicobacter pylori* BM012A (CP006888.1) | *Helicobacter pylori* Cuz20 (CP002076.1) |
| *Helicobacter pylori* ELS37 (CP002953.1) | *Helicobacter pylori* Gambia94/24 (CP002332.1) |
| *Helicobacter pylori* India7 (CP002331.1) | *Helicobacter pylori* Lithuania75 (CP002334.1) |
| *Helicobacter pylori* PeCan4 (CP002074.1) | *Helicobacter pylori* SJM180 (CP002073.1) |
| *Helicobacter pylori* SNT49 (CP002983.1) | *Helicobacter pylori* SouthAfrica20 (CP006691.1) |
| *Helicobacter pylori* SouthAfrica7  (CP002336.1) | *Helicobacter pylori* UM066 (CP005493.3) |
| *Helicobacter pylori* UM298 (CP006610.2) | *Helicobacter pylori* B8 (FN598874.1) |
| *Helicobacter pylori* v225d (CP001582.1) |  |

^a^IDs in brackets are accessions.

**Table S2.** Number of peptides digested from the generated protein amino acid sequences, number of peptides similar to the original F16 sequences, and reproduction percentage ratio for F16 sequences across various random branch generations.

| Leaves^a^ | Branch^a^ | Generation^a^ | Method^b^ | #of protein | # of non-redundant peptides^c^ | # of peptides in F16^d^ | %cover^e^ |
| --- | --- | --- | --- | --- | --- | --- | --- |
| 3 | 50 | 10 | strict | 986,137 | 1,618,632 | 30,549 | 94.3 |
| 3 | 50 | 10 | order | 1,724,176 | 2,713,423 | 30,721 | 94.8 |
| 3 | 50 | 10 | nearest | 1,717,903 | 2,663,548 | 30,705 | 94.8 |

^a^Leaves, Branch, and Generation are as described in Figure 3

^b^see Section 2.1.4

^c^The number of peptides digested from the full length of the generated amino acid sequence with allowed miss-cleavage is 1.

^d^The number of peptides which the identical peptides to in the peptides digested from the amino acid sequences of the original F16 strain.

^e^The ratio of the “In F16” to the number of peptides from the original F16 strain. The number of digested peptides of the original F16 strain is 32,398.

**Table S3.** Number of proteins and non-redundant digested peptides in the amino acid sequence database for F16, pylori29, and rb3_5, rb3_50.

| Branch (B) axis |  |  |  |  |
| --- | --- | --- | --- | --- |
| Data set | #of protein^a^ | # of non-redundant peptides^a^ | # of peptides in F16^a^ | %cover^a^ |
| F16 | 1,500 | 32,398 | 32,398 |  |
| pylori29 | 44,708 | 161,550 | 30,185 | 93.2 |
| rb3_50_mk10 | 1,717,903 | 2,663,548 | 30,705 | 94.8 |
| rb10_50_mk10 | 1,844,397 | 2,843,883 | 30,730 | 94.9 |
| rb15_50_mk10 | 1,842,967 | 2,820,416 | 30,723 | 94.8 |
|  |  |  |  |  |
| Leaves (L) axis |  |  |  |  |
|  | #of protein | # of non-redundant peptides | # of peptides in F16 | %cover |
| F16 | 1,500 | 32,398 | 32,398 |  |
| pylori29 | 44,708 | 161,550 | 30,185 | 93.2 |
| rb3_5_mk10 | 428,965 | 694,562 | 30,429 | 93.9 |
| rb3_50_mk10 | 1,717,903 | 2,663,548 | 30,705 | 94.8 |
| rb3_100_mk10 | 2,453,237 | 3,872,209 | 30,771 | 95.0 |
|  |  |  |  |  |
| Generation (N) axis |  |  |  |  |
|  | #of protein | # of non-redundant peptides | # of peptides in F16 | %cover |
| F16 | 1,500 | 32,398 | 32,398 |  |
| pylori29 | 44,708 | 161,550 | 30,185 | 93.2 |
| rb3_5_mk10 | 428,965 | 694,562 | 30,429 | 93.9 |
| rb3_50_mk10 | 1,717,903 | 2,663,548 | 30,705 | 94.8 |
| rb3_50_mk100 | 16,621,417 | 14,216,229 | 31,246 | 96.4 |
| rb3_50_mk500 | 80,942,290 | 39,880,351 | 31,587 | 97.5 |
| rb3_50_mk1000 | 158,080,270 | 60,248,709 | 31,663 | 97.7 |
| rb3_50_mk5000 | 654,241,091 | 82,819,615 | 31,778 | 98.1 |

^a^These mean the same as Table S2.

**Table S4.** Number of trypsin digested peptide with miss cleavage is 0, the number of peptides matcnhed with those of F16 strain, and the percentage for the SIHUMIx and SIHUMIx_rb8_10_mk10. The number of digested peptides of F16 is 32,398.

|  | The number of digested peptides^a^ | The number of peptides matched with F16^b^ |
| --- | --- | --- |
| SIHUMIx | 577,233 | 1,866 (5.8%) |
| SIHUMIx_rb8_10_mk10 | 3,048,575 | 4,583 (14.2%) |

^a^ The number of trypsin digested peptide with miss cleavage is 0.

^b^ The numbers in parentheses are the percentages relative to the number of digested peptides (32,398) from F16 strai

**Dataset S1.** Input parameters for MaxQuant calculations of raw files for the *Helicobacter* *pylori* F16 strain with the F16 database.

<?xml version="1.0" encoding="utf-8"?>

<MaxQuantParams xmlns:xsd="http://www.w3.org/2001/XMLSchema" xmlns:xsi="http://www.w3.org/2001/XMLSchema-instance">

<fastaFiles>

<FastaFileInfo>

<fastaFilePath>fasta file name</fastaFilePath>

<identifierParseRule>>([^\s]*)</identifierParseRule>

<descriptionParseRule>>(.*)</descriptionParseRule>

<taxonomyParseRule></taxonomyParseRule>

<variationParseRule></variationParseRule>

<modificationParseRule></modificationParseRule>

<taxonomyId></taxonomyId>

</FastaFileInfo>

</fastaFiles>

<fastaFilesProteogenomics>

</fastaFilesProteogenomics>

<fastaFilesFirstSearch>

</fastaFilesFirstSearch>

<fixedSearchFolder></fixedSearchFolder>

<andromedaCacheSize>350000</andromedaCacheSize>

<advancedRatios>True</advancedRatios>

<pvalThres>0.005</pvalThres>

<rtShift>False</rtShift>

<separateLfq>False</separateLfq>

<lfqStabilizeLargeRatios>True</lfqStabilizeLargeRatios>

<lfqRequireMsms>True</lfqRequireMsms>

<lfqBayesQuant>False</lfqBayesQuant>

<decoyMode>revert</decoyMode>

<includeContaminants>True</includeContaminants>

<maxPeptideMass>4600</maxPeptideMass>

<epsilonMutationScore>True</epsilonMutationScore>

<mutatedPeptidesSeparately>True</mutatedPeptidesSeparately>

<proteogenomicPeptidesSeparately>True</proteogenomicPeptidesSeparately>

<minDeltaScoreUnmodifiedPeptides>0</minDeltaScoreUnmodifiedPeptides>

<minDeltaScoreModifiedPeptides>6</minDeltaScoreModifiedPeptides>

<minScoreUnmodifiedPeptides>0</minScoreUnmodifiedPeptides>

<minScoreModifiedPeptides>40</minScoreModifiedPeptides>

<secondPeptide>True</secondPeptide>

<matchBetweenRuns>False</matchBetweenRuns>

<matchUnidentifiedFeatures>False</matchUnidentifiedFeatures>

<matchBetweenRunsFdr>False</matchBetweenRunsFdr>

<dependentPeptides>False</dependentPeptides>

<dependentPeptideFdr>0</dependentPeptideFdr>

<dependentPeptideMassBin>0</dependentPeptideMassBin>

<dependentPeptidesBetweenRuns>False</dependentPeptidesBetweenRuns>

<dependentPeptidesWithinExperiment>False</dependentPeptidesWithinExperiment>

<dependentPeptidesWithinParameterGroup>False</dependentPeptidesWithinParameterGroup>

<dependentPeptidesRestrictFractions>False</dependentPeptidesRestrictFractions>

<dependentPeptidesFractionDifference>0</dependentPeptidesFractionDifference>

<ibaq>False</ibaq>

<top3>False</top3>

<independentEnzymes>False</independentEnzymes>

<useDeltaScore>False</useDeltaScore>

<splitProteinGroupsByTaxonomy>True</splitProteinGroupsByTaxonomy>

<taxonomyLevel>Species</taxonomyLevel>

<avalon>False</avalon>

<nModColumns>3</nModColumns>

<ibaqLogFit>False</ibaqLogFit>

<ibaqChargeNormalization>False</ibaqChargeNormalization>

<razorProteinFdr>True</razorProteinFdr>

<deNovoSequencing>False</deNovoSequencing>

<deNovoVarMods>False</deNovoVarMods>

<deNovoCompleteSequence>False</deNovoCompleteSequence>

<deNovoCalibratedMasses>False</deNovoCalibratedMasses>

<deNovoMaxIterations>0</deNovoMaxIterations>

<deNovoProteaseReward>0</deNovoProteaseReward>

<deNovoProteaseRewardTof>0</deNovoProteaseRewardTof>

<deNovoAgPenalty>0</deNovoAgPenalty>

<deNovoGgPenalty>0</deNovoGgPenalty>

<deNovoUseComplementScore>True</deNovoUseComplementScore>

<deNovoUseProteaseScore>True</deNovoUseProteaseScore>

<deNovoUseWaterLossScore>True</deNovoUseWaterLossScore>

<deNovoUseAmmoniaLossScore>True</deNovoUseAmmoniaLossScore>

<deNovoUseA2Score>True</deNovoUseA2Score>

<deNovoMassClusterTolDa>0</deNovoMassClusterTolDa>

<deNovoScalingFactor>0</deNovoScalingFactor>

<massDifferenceSearch>False</massDifferenceSearch>

<isotopeCalc>False</isotopeCalc>

<minPepLen>7</minPepLen>

<psmFdrCrosslink>0.01</psmFdrCrosslink>

<peptideFdr>0.01</peptideFdr>

<proteinFdr>0.01</proteinFdr>

<siteFdr>0.01</siteFdr>

<minPeptideLengthForUnspecificSearch>8</minPeptideLengthForUnspecificSearch>

<maxPeptideLengthForUnspecificSearch>25</maxPeptideLengthForUnspecificSearch>

<useNormRatiosForOccupancy>True</useNormRatiosForOccupancy>

<minPeptides>1</minPeptides>

<minRazorPeptides>1</minRazorPeptides>

<minUniquePeptides>0</minUniquePeptides>

<useCounterparts>False</useCounterparts>

<advancedSiteIntensities>True</advancedSiteIntensities>

<customProteinQuantification>False</customProteinQuantification>

<customProteinQuantificationFile></customProteinQuantificationFile>

<minRatioCount>2</minRatioCount>

<restrictProteinQuantification>True</restrictProteinQuantification>

<restrictMods>

<string>Oxidation (M)</string>

<string>Acetyl (Protein N-term)</string>

</restrictMods>

<matchingTimeWindow>0</matchingTimeWindow>

<matchingIonMobilityWindow>0</matchingIonMobilityWindow>

<alignmentTimeWindow>0</alignmentTimeWindow>

<alignmentIonMobilityWindow>0</alignmentIonMobilityWindow>

<numberOfCandidatesMsms>15</numberOfCandidatesMsms>

<compositionPrediction>0</compositionPrediction>

<quantMode>1</quantMode>

<massDifferenceMods>

</massDifferenceMods>

<mainSearchMaxCombinations>200</mainSearchMaxCombinations>

<writeMsScansTable>False</writeMsScansTable>

<writeMsmsScansTable>True</writeMsmsScansTable>

<writePasefMsmsScansTable>True</writePasefMsmsScansTable>

<writeAccumulatedMsmsScansTable>True</writeAccumulatedMsmsScansTable>

<writeMs3ScansTable>True</writeMs3ScansTable>

<writeAllPeptidesTable>False</writeAllPeptidesTable>

<writeMzRangeTable>True</writeMzRangeTable>

<writeDiaFragmentTable>False</writeDiaFragmentTable>

<writeDiaFragmentQuantTable>False</writeDiaFragmentQuantTable>

<writeMzTab>False</writeMzTab>

<writeSdrf>False</writeSdrf>

<disableMd5>False</disableMd5>

<cacheBinInds>True</cacheBinInds>

<etdIncludeB>False</etdIncludeB>

<ms2PrecursorShift>0</ms2PrecursorShift>

<complementaryIonPpm>20</complementaryIonPpm>

<variationParseRule></variationParseRule>

<variationMode>none</variationMode>

<useSeriesReporters>False</useSeriesReporters>

<name>セッション1</name>

<maxQuantVersion>2.5.2.0</maxQuantVersion>

<pluginFolder></pluginFolder>

<numThreads>8</numThreads>

<emailAddress></emailAddress>

<smtpHost></smtpHost>

<emailFromAddress></emailFromAddress>

<fullMinMz>-1.7976931348623157E+308</fullMinMz>

<fullMaxMz>1.7976931348623157E+308</fullMaxMz>

<sendEmail>False</sendEmail>

<ionCountIntensities>False</ionCountIntensities>

<verboseColumnHeaders>False</verboseColumnHeaders>

<calcPeakProperties>False</calcPeakProperties>

<showCentroidMassDifferences>False</showCentroidMassDifferences>

<showIsotopeMassDifferences>False</showIsotopeMassDifferences>

<profilePerformance>False</profilePerformance>

<filePaths>

<string>C:\JobRequest\JobObjects\bin\binExecDir\raw\ManualInputFile_pylori_F16\151005sm_F16-1.raw</string>

<string>C:\JobRequest\JobObjects\bin\binExecDir\raw\ManualInputFile_pylori_F16\151005sm_F16-2.raw</string>

<string>C:\JobRequest\JobObjects\bin\binExecDir\raw\ManualInputFile_pylori_F16\151005sm_F16-3.raw</string>

<string>C:\JobRequest\JobObjects\bin\binExecDir\raw\ManualInputFile_pylori_F16\151005sm_F16-4.raw</string>

<string>C:\JobRequest\JobObjects\bin\binExecDir\raw\ManualInputFile_pylori_F16\151005sm_F16-5.raw</string>

</filePaths>

<experiments>

<string></string>

<string></string>

<string></string>

<string></string>

<string></string>

</experiments>

<fractions>

<short>32767</short>

<short>32767</short>

<short>32767</short>

<short>32767</short>

<short>32767</short>

</fractions>

<ptms>

<boolean>False</boolean>

<boolean>False</boolean>

<boolean>False</boolean>

<boolean>False</boolean>

<boolean>False</boolean>

</ptms>

<paramGroupIndices>

<int>0</int>

<int>0</int>

<int>0</int>

<int>0</int>

<int>0</int>

</paramGroupIndices>

<referenceChannel>

<string></string>

<string></string>

<string></string>

<string></string>

<string></string>

</referenceChannel>

<lfqTopNPeptides>0</lfqTopNPeptides>

<diaJoinPrecChargesForLfq>False</diaJoinPrecChargesForLfq>

<diaFragChargesForQuant>1</diaFragChargesForQuant>

<gridSpacing>0.7</gridSpacing>

<proteinGroupingFile></proteinGroupingFile>

<simplePepCalculation>False</simplePepCalculation>

<useAndromeda20>False</useAndromeda20>

<useAndromeda20DefaultModel>False</useAndromeda20DefaultModel>

<andromeda20AltModelPath></andromeda20AltModelPath>

<intensityPredictionFolder></intensityPredictionFolder>

<encoding>0</encoding>

<parameterGroups>

<parameterGroup>

<andromeda20AltModelPath></andromeda20AltModelPath>

<andromeda20DefaultModel>False</andromeda20DefaultModel>

<useAndromeda20>False</useAndromeda20>

<msInstrument>0</msInstrument>

<maxCharge>7</maxCharge>

<minPeakLen>2</minPeakLen>

<diaMinPeakLen>1</diaMinPeakLen>

<useMs1Centroids>False</useMs1Centroids>

<useMs2Centroids>False</useMs2Centroids>

<cutPeaks>True</cutPeaks>

<gapScans>1</gapScans>

<minTime>NaN</minTime>

<maxTime>NaN</maxTime>

<matchType>MatchFromAndTo</matchType>

<intensityDetermination>0</intensityDetermination>

<centroidMatchTol>8</centroidMatchTol>

<centroidMatchTolInPpm>True</centroidMatchTolInPpm>

<centroidHalfWidth>35</centroidHalfWidth>

<centroidHalfWidthInPpm>True</centroidHalfWidthInPpm>

<valleyFactor>1.4</valleyFactor>

<isotopeValleyFactor>1.2</isotopeValleyFactor>

<advancedPeakSplitting>False</advancedPeakSplitting>

<intensityThresholdMs1Dda>0</intensityThresholdMs1Dda>

<intensityThresholdMs1Dia>0</intensityThresholdMs1Dia>

<intensityThresholdMs2>0</intensityThresholdMs2>

<labelMods>

<string></string>

</labelMods>

<lcmsRunType>Standard</lcmsRunType>

<reQuantify>False</reQuantify>

<lfqMode>0</lfqMode>

<lfqNormClusterSize>80</lfqNormClusterSize>

<lfqMinEdgesPerNode>3</lfqMinEdgesPerNode>

<lfqAvEdgesPerNode>6</lfqAvEdgesPerNode>

<lfqMaxFeatures>100000</lfqMaxFeatures>

<neucodeMaxPpm>0</neucodeMaxPpm>

<neucodeResolution>0</neucodeResolution>

<neucodeResolutionInMda>False</neucodeResolutionInMda>

<neucodeInSilicoLowRes>False</neucodeInSilicoLowRes>

<fastLfq>True</fastLfq>

<lfqRestrictFeatures>False</lfqRestrictFeatures>

<lfqMinRatioCount>2</lfqMinRatioCount>

<lfqMinRatioCountDia>2</lfqMinRatioCountDia>

<lfqPrioritizeMs1Dia>True</lfqPrioritizeMs1Dia>

<maxLabeledAa>0</maxLabeledAa>

<maxNmods>5</maxNmods>

<maxMissedCleavages>2</maxMissedCleavages>

<multiplicity>1</multiplicity>

<enzymeMode>0</enzymeMode>

<complementaryReporterType>0</complementaryReporterType>

<reporterNormalization>0</reporterNormalization>

<neucodeIntensityMode>0</neucodeIntensityMode>

<fixedModifications>

<string>Carbamidomethyl (C)</string>

</fixedModifications>

<enzymes>

<string>Trypsin/P</string>

</enzymes>

<enzymesFirstSearch>

</enzymesFirstSearch>

<enzymeModeFirstSearch>0</enzymeModeFirstSearch>

<useEnzymeFirstSearch>False</useEnzymeFirstSearch>

<useVariableModificationsFirstSearch>False</useVariableModificationsFirstSearch>

<variableModifications>

<string>Oxidation (M)</string>

<string>Acetyl (Protein N-term)</string>

</variableModifications>

<useMultiModification>False</useMultiModification>

<multiModifications>

</multiModifications>

<isobaricLabels>

</isobaricLabels>

<neucodeLabels>

</neucodeLabels>

<variableModificationsFirstSearch>

</variableModificationsFirstSearch>

<hasAdditionalVariableModifications>False</hasAdditionalVariableModifications>

<additionalVariableModifications>

</additionalVariableModifications>

<additionalVariableModificationProteins>

</additionalVariableModificationProteins>

<doMassFiltering>True</doMassFiltering>

<firstSearchTol>20</firstSearchTol>

<mainSearchTol>4.5</mainSearchTol>

<searchTolInPpm>True</searchTolInPpm>

<isotopeMatchTol>2</isotopeMatchTol>

<isotopeMatchTolInPpm>True</isotopeMatchTolInPpm>

<isotopeTimeCorrelation>0.6</isotopeTimeCorrelation>

<theorIsotopeCorrelation>0.6</theorIsotopeCorrelation>

<checkMassDeficit>True</checkMassDeficit>

<recalibrationInPpm>True</recalibrationInPpm>

<intensityDependentCalibration>False</intensityDependentCalibration>

<minScoreForCalibration>70</minScoreForCalibration>

<matchLibraryFile>False</matchLibraryFile>

<libraryFile></libraryFile>

<matchLibraryMassTolPpm>0</matchLibraryMassTolPpm>

<matchLibraryTimeTolMin>0</matchLibraryTimeTolMin>

<matchLabelTimeTolMin>0</matchLabelTimeTolMin>

<reporterMassTolerance>NaN</reporterMassTolerance>

<reporterPif>NaN</reporterPif>

<filterPif>False</filterPif>

<reporterFraction>NaN</reporterFraction>

<reporterBasePeakRatio>NaN</reporterBasePeakRatio>

<timsHalfWidth>0</timsHalfWidth>

<timsStep>0</timsStep>

<timsResolution>0</timsResolution>

<timsMinMsmsIntensity>0</timsMinMsmsIntensity>

<timsRemovePrecursor>True</timsRemovePrecursor>

<timsIsobaricLabels>False</timsIsobaricLabels>

<timsCollapseMsms>True</timsCollapseMsms>

<crossLinkingType>0</crossLinkingType>

<crossLinker></crossLinker>

<minMatchXl>3</minMatchXl>

<minPairedPepLenXl>6</minPairedPepLenXl>

<minScoreDipeptide>40</minScoreDipeptide>

<minScoreMonopeptide>0</minScoreMonopeptide>

<minScorePartialCross>10</minScorePartialCross>

<crosslinkOnlyIntraProtein>False</crosslinkOnlyIntraProtein>

<crosslinkIntensityBasedPrecursor>True</crosslinkIntensityBasedPrecursor>

<isHybridPrecDetermination>False</isHybridPrecDetermination>

<topXcross>3</topXcross>

<doesSeparateInterIntraProteinCross>False</doesSeparateInterIntraProteinCross>

<crosslinkMaxMonoUnsaturated>0</crosslinkMaxMonoUnsaturated>

<crosslinkMaxMonoSaturated>0</crosslinkMaxMonoSaturated>

<crosslinkMaxDiUnsaturated>0</crosslinkMaxDiUnsaturated>

<crosslinkMaxDiSaturated>0</crosslinkMaxDiSaturated>

<crosslinkModifications>

</crosslinkModifications>

<crosslinkFastaFiles>

</crosslinkFastaFiles>

<crosslinkSites>

</crosslinkSites>

<crosslinkNetworkFiles>

</crosslinkNetworkFiles>

<crosslinkMode></crosslinkMode>

<peakRefinement>False</peakRefinement>

<peakRefinementCrosslinking>False</peakRefinementCrosslinking>

<isobaricSumOverWindow>True</isobaricSumOverWindow>

<isobaricWeightExponent>0.75</isobaricWeightExponent>

<collapseMsmsOnIsotopePatterns>False</collapseMsmsOnIsotopePatterns>

<diaLibraryType>0</diaLibraryType>

<diaLibraryPaths>

</diaLibraryPaths>

<diaEvidencePaths>

</diaEvidencePaths>

<diaMsmsPaths>

</diaMsmsPaths>

<diaLabelIndsForLibraryMatch>

</diaLabelIndsForLibraryMatch>

<diaInitialPrecMassTolPpm>20</diaInitialPrecMassTolPpm>

<diaInitialFragMassTolPpm>20</diaInitialFragMassTolPpm>

<diaCorrThresholdFeatureClustering>0.85</diaCorrThresholdFeatureClustering>

<diaPrecTolPpmFeatureClustering>2</diaPrecTolPpmFeatureClustering>

<diaFragTolPpmFeatureClustering>2</diaFragTolPpmFeatureClustering>

<diaScoreN>12</diaScoreN>

<diaScoreNAdditional>5</diaScoreNAdditional>

<diaMinScore>1.99</diaMinScore>

<diaXgBoostBaseScore>0.4</diaXgBoostBaseScore>

<diaXgBoostSubSample>0.9</diaXgBoostSubSample>

<centroidPosition>0</centroidPosition>

<diaQuantMethod>7</diaQuantMethod>

<diaFeatureQuantMethod>2</diaFeatureQuantMethod>

<lfqNormType>1</lfqNormType>

<diaTopNForQuant>12</diaTopNForQuant>

<diaTopNCorrelationForQuant>7</diaTopNCorrelationForQuant>

<diaFragmentCorrelationForQuant>0.78</diaFragmentCorrelationForQuant>

<diaMinMsmsIntensityForQuant>0</diaMinMsmsIntensityForQuant>

<diaTopMsmsIntensityQuantileForQuant>0.85</diaTopMsmsIntensityQuantileForQuant>

<diaMinFragmentOverlapScore>0</diaMinFragmentOverlapScore>

<diaMinPrecursorScore>0</diaMinPrecursorScore>

<diaUseProfileCorrelation>False</diaUseProfileCorrelation>

<diaMinPrecProfileCorrelation>0</diaMinPrecProfileCorrelation>

<diaMinFragProfileCorrelation>0</diaMinFragProfileCorrelation>

<diaXgBoostMinChildWeight>9</diaXgBoostMinChildWeight>

<diaXgBoostMaximumTreeDepth>12</diaXgBoostMaximumTreeDepth>

<diaXgBoostEstimators>580</diaXgBoostEstimators>

<diaXgBoostGamma>0.9</diaXgBoostGamma>

<diaXgBoostMaxDeltaStep>3</diaXgBoostMaxDeltaStep>

<diaGlobalMl>True</diaGlobalMl>

<diaAdaptiveMassAccuracy>False</diaAdaptiveMassAccuracy>

<diaMassWindowFactor>3.3</diaMassWindowFactor>

<diaNoMl>False</diaNoMl>

<diaPermuteRt>False</diaPermuteRt>

<diaPermuteCcs>False</diaPermuteCcs>

<diaBackgroundSubtraction>False</diaBackgroundSubtraction>

<diaBackgroundSubtractionQuantile>0.5</diaBackgroundSubtractionQuantile>

<diaBackgroundSubtractionFactor>4</diaBackgroundSubtractionFactor>

<diaLfqRatioType>0</diaLfqRatioType>

<diaTransferQvalue>0.3</diaTransferQvalue>

<diaTransferQvalueBetweenLabels>0.01</diaTransferQvalueBetweenLabels>

<diaTransferQvalueBetweenFractions>0.01</diaTransferQvalueBetweenFractions>

<diaTransferQvalueBetweenFaims>0.01</diaTransferQvalueBetweenFaims>

<diaOnlyIsosForRecal>True</diaOnlyIsosForRecal>

<diaMinPeaks>5</diaMinPeaks>

<diaUseFragIntensForMl>False</diaUseFragIntensForMl>

<diaUseFragMassesForMl>False</diaUseFragMassesForMl>

<diaMaxTrainInstances>500000</diaMaxTrainInstances>

<diaMaxFragmentCharge>3</diaMaxFragmentCharge>

<diaAdaptiveMlScoring>False</diaAdaptiveMlScoring>

<diaDynamicScoringMaxInstances>25000</diaDynamicScoringMaxInstances>

<diaMaxPrecursorMz>0</diaMaxPrecursorMz>

<diaHardRtFilter>True</diaHardRtFilter>

<diaConvertLibraryCharge2Fragments>False</diaConvertLibraryCharge2Fragments>

<diaChargeNormalizationLibrary>True</diaChargeNormalizationLibrary>

<diaChargeNormalizationSample>True</diaChargeNormalizationSample>

<diaDeleteIntermediateResults>True</diaDeleteIntermediateResults>

<diaScoreWeightScanIndex>-1</diaScoreWeightScanIndex>

<diaScoreWeightScanValue>1</diaScoreWeightScanValue>

<diaNumNonleadingMatches>1</diaNumNonleadingMatches>

<diaUseDefaultFragmentModel>True</diaUseDefaultFragmentModel>

<diaAltFragmentModelPath></diaAltFragmentModelPath>

<diaUseDefaultRtModel>True</diaUseDefaultRtModel>

<diaAltRtModelPath></diaAltRtModelPath>

<diaUseDefaultCcsModel>True</diaUseDefaultCcsModel>

<diaAltCcsModelPath></diaAltCcsModelPath>

<diaBatchProcessing>True</diaBatchProcessing>

<diaBatchSize>25</diaBatchSize>

<diaFirstBatch>-1</diaFirstBatch>

<diaLastBatch>-1</diaLastBatch>

<diaOnlyPreprocess>False</diaOnlyPreprocess>

<diaMultiplexQuantMethod>1</diaMultiplexQuantMethod>

<diaOnlyPostprocess>False</diaOnlyPostprocess>

<diaRequirePrecursor>False</diaRequirePrecursor>

<diaFuturePeptides>False</diaFuturePeptides>

<diaOverrideRtWithPrediction>False</diaOverrideRtWithPrediction>

<diaMaxModifications>0</diaMaxModifications>

<diaMaxPositionings>0</diaMaxPositionings>

<diaUseProbScore>False</diaUseProbScore>

<diaProbScoreP>0.055</diaProbScoreP>

<diaProbScoreG>1.2</diaProbScoreG>

<diaProbScoreStep>0.1</diaProbScoreStep>

<isM2FragTypeOverride>False</isM2FragTypeOverride>

<ms2FragTypeOverride>0</ms2FragTypeOverride>

<classicLfqForSingleShots>True</classicLfqForSingleShots>

<sequenceBasedModifier>False</sequenceBasedModifier>

<diaRtFromSamplesForExport>True</diaRtFromSamplesForExport>

<diaCcsFromSamplesForExport>True</diaCcsFromSamplesForExport>

<diaLibraryExport>0</diaLibraryExport>

<diaUseApexWeightsForPtmLoc>False</diaUseApexWeightsForPtmLoc>

<diaSecondScoreForMultiplex>True</diaSecondScoreForMultiplex>

<diaBestPrecursorChargeForQuant>False</diaBestPrecursorChargeForQuant>

<diaBestPrecursorMassRangeForQuant>False</diaBestPrecursorMassRangeForQuant>

<diaBestPrecursorIntensityForQuant>False</diaBestPrecursorIntensityForQuant>

<diaPtmLocMethod>0</diaPtmLocMethod>

</parameterGroup>

</parameterGroups>

<msmsParamsArray>

<msmsParams>

<Name>FTMS</Name>

<MatchTolerance>20</MatchTolerance>

<MatchToleranceInPpm>True</MatchToleranceInPpm>

<DeisotopeTolerance>7</DeisotopeTolerance>

<DeisotopeToleranceInPpm>True</DeisotopeToleranceInPpm>

<DeNovoTolerance>25</DeNovoTolerance>

<DeNovoToleranceInPpm>True</DeNovoToleranceInPpm>

<Deisotope>True</Deisotope>

<Topx>12</Topx>

<TopxInterval>100</TopxInterval>

<HigherCharges>True</HigherCharges>

<IncludeWater>True</IncludeWater>

<IncludeAmmonia>True</IncludeAmmonia>

<IncludeWaterCross>False</IncludeWaterCross>

<IncludeAmmoniaCross>False</IncludeAmmoniaCross>

<DependentLosses>True</DependentLosses>

<Recalibration>False</Recalibration>

</msmsParams>

<msmsParams>

<Name>ITMS</Name>

<MatchTolerance>0.5</MatchTolerance>

<MatchToleranceInPpm>False</MatchToleranceInPpm>

<DeisotopeTolerance>0.15</DeisotopeTolerance>

<DeisotopeToleranceInPpm>False</DeisotopeToleranceInPpm>

<DeNovoTolerance>0.5</DeNovoTolerance>

<DeNovoToleranceInPpm>False</DeNovoToleranceInPpm>

<Deisotope>False</Deisotope>

<Topx>8</Topx>

<TopxInterval>100</TopxInterval>

<HigherCharges>True</HigherCharges>

<IncludeWater>True</IncludeWater>

<IncludeAmmonia>True</IncludeAmmonia>

<IncludeWaterCross>False</IncludeWaterCross>

<IncludeAmmoniaCross>False</IncludeAmmoniaCross>

<DependentLosses>True</DependentLosses>

<Recalibration>False</Recalibration>

</msmsParams>

<msmsParams>

<Name>TOF</Name>

<MatchTolerance>25</MatchTolerance>

<MatchToleranceInPpm>True</MatchToleranceInPpm>

<DeisotopeTolerance>0.01</DeisotopeTolerance>

<DeisotopeToleranceInPpm>False</DeisotopeToleranceInPpm>

<DeNovoTolerance>25</DeNovoTolerance>

<DeNovoToleranceInPpm>True</DeNovoToleranceInPpm>

<Deisotope>True</Deisotope>

<Topx>16</Topx>

<TopxInterval>100</TopxInterval>

<HigherCharges>True</HigherCharges>

<IncludeWater>True</IncludeWater>

<IncludeAmmonia>True</IncludeAmmonia>

<IncludeWaterCross>False</IncludeWaterCross>

<IncludeAmmoniaCross>False</IncludeAmmoniaCross>

<DependentLosses>True</DependentLosses>

<Recalibration>False</Recalibration>

</msmsParams>

<msmsParams>

<Name>UNKNOWN</Name>

<MatchTolerance>20</MatchTolerance>

<MatchToleranceInPpm>True</MatchToleranceInPpm>

<DeisotopeTolerance>7</DeisotopeTolerance>

<DeisotopeToleranceInPpm>True</DeisotopeToleranceInPpm>

<DeNovoTolerance>25</DeNovoTolerance>

<DeNovoToleranceInPpm>True</DeNovoToleranceInPpm>

<Deisotope>True</Deisotope>

<Topx>12</Topx>

<TopxInterval>100</TopxInterval>

<HigherCharges>True</HigherCharges>

<IncludeWater>True</IncludeWater>

<IncludeAmmonia>True</IncludeAmmonia>

<IncludeWaterCross>False</IncludeWaterCross>

<IncludeAmmoniaCross>False</IncludeAmmoniaCross>

<DependentLosses>True</DependentLosses>

<Recalibration>False</Recalibration>

</msmsParams>

</msmsParamsArray>

<fragmentationParamsArray>

<fragmentationParams>

<Name>HCD</Name>

<UseIntensityPrediction>False</UseIntensityPrediction>

<UseSequenceBasedModifier>False</UseSequenceBasedModifier>

<InternalFragments>False</InternalFragments>

<InternalFragmentWeight>1</InternalFragmentWeight>

<InternalFragmentAas>KRH</InternalFragmentAas>

</fragmentationParams>

<fragmentationParams>

<Name>CID</Name>

<UseIntensityPrediction>False</UseIntensityPrediction>

<UseSequenceBasedModifier>False</UseSequenceBasedModifier>

<InternalFragments>False</InternalFragments>

<InternalFragmentWeight>1</InternalFragmentWeight>

<InternalFragmentAas>KRH</InternalFragmentAas>

</fragmentationParams>

<fragmentationParams>

<Name>ETD</Name>

<UseIntensityPrediction>False</UseIntensityPrediction>

<UseSequenceBasedModifier>False</UseSequenceBasedModifier>

<InternalFragments>False</InternalFragments>

<InternalFragmentWeight>1</InternalFragmentWeight>

<InternalFragmentAas>KRH</InternalFragmentAas>

</fragmentationParams>

<fragmentationParams>

<Name>PQD</Name>

<UseIntensityPrediction>False</UseIntensityPrediction>

<UseSequenceBasedModifier>False</UseSequenceBasedModifier>

<InternalFragments>False</InternalFragments>

<InternalFragmentWeight>1</InternalFragmentWeight>

<InternalFragmentAas>KRH</InternalFragmentAas>

</fragmentationParams>

<fragmentationParams>

<Name>ETHCD</Name>

<UseIntensityPrediction>False</UseIntensityPrediction>

<UseSequenceBasedModifier>False</UseSequenceBasedModifier>

<InternalFragments>False</InternalFragments>

<InternalFragmentWeight>1</InternalFragmentWeight>

<InternalFragmentAas>KRH</InternalFragmentAas>

</fragmentationParams>

<fragmentationParams>

<Name>ETCID</Name>

<UseIntensityPrediction>False</UseIntensityPrediction>

<UseSequenceBasedModifier>False</UseSequenceBasedModifier>

<InternalFragments>False</InternalFragments>

<InternalFragmentWeight>1</InternalFragmentWeight>

<InternalFragmentAas>KRH</InternalFragmentAas>

</fragmentationParams>

<fragmentationParams>

<Name>UVPD</Name>

<UseIntensityPrediction>False</UseIntensityPrediction>

<UseSequenceBasedModifier>False</UseSequenceBasedModifier>

<InternalFragments>False</InternalFragments>

<InternalFragmentWeight>1</InternalFragmentWeight>

<InternalFragmentAas>KRH</InternalFragmentAas>

</fragmentationParams>

<fragmentationParams>

<Name>Unknown</Name>

<UseIntensityPrediction>False</UseIntensityPrediction>

<UseSequenceBasedModifier>False</UseSequenceBasedModifier>

<InternalFragments>False</InternalFragments>

<InternalFragmentWeight>1</InternalFragmentWeight>

<InternalFragmentAas>KRH</InternalFragmentAas>

</fragmentationParams>

</fragmentationParamsArray>

</MaxQuantParams>

**Dataset S2.** Input parameters for Comet calculations of raw files for the *Helicobacter* *pylori* F16 strain with the F16 database.

# comet_version 2019.01 rev. 5

# Comet MS/MS search engine parameters file.

# Everything following the '#' symbol is treated as a comment.

database_name = fasta_file_name.fasta

decoy_search = 0

peff_format = 0

peff_obo =

num_threads = 8

peptide_mass_tolerance = 10

peptide_mass_units = 2

mass_type_parent = 1

mass_type_fragment = 1

precursor_tolerance_type = 1

isotope_error = 0

search_enzyme_number = 12

num_enzyme_termini = 2

allowed_missed_cleavage = 0

variable_mod01 = 15.994915 M 0 5 -1 0 0 63.998285

variable_mod02 = 0.0 X 0 3 -1 0 0 0.0

variable_mod03 = 0.0 X 0 3 -1 0 0 0.0

variable_mod04 = 0.0 X 0 3 -1 0 0 0.0

variable_mod05 = 0.0 X 0 3 -1 0 0 0.0

variable_mod06 = 0.0 X 0 3 -1 0 0 0.0

variable_mod07 = 0.0 X 0 3 -1 0 0 0.0

variable_mod08 = 0.0 X 0 3 -1 0 0 0.0

variable_mod09 = 0.0 X 0 3 -1 0 0 0.0

max_variable_mods_in_peptide = 5

require_variable_mod = 0

fragment_bin_tol = 0.02

fragment_bin_offset = 0.0

theoretical_fragment_ions = 0

use_A_ions = 0

use_B_ions = 1

use_C_ions = 0

use_X_ions = 0

use_Y_ions = 1

use_Z_ions = 0

use_NL_ions = 1

output_sqtstream = 0

output_sqtfile = 0

output_txtfile = 0

output_pepxmlfile = 1

output_percolatorfile = 1

print_expect_score = 1

num_output_lines = 5

show_fragment_ions = 0

sample_enzyme_number = 12

scan_range = 0 0

precursor_charge = 0 0

override_charge = 0

ms_level = 2

activation_method = ALL

digest_mass_range = 600.0 5000.0

peptide_length_range = 7 63

num_results = 100

max_duplicate_proteins = 20

skip_researching = 1

max_fragment_charge = 3

max_precursor_charge = 6

nucleotide_reading_frame = 0

clip_nterm_methionine = 0

spectrum_batch_size = 20000

decoy_prefix = ###REV###

equal_I_and_L = 1

output_suffix =

mass_offsets =

precursor_NL_ions =

minimum_peaks = 10

minimum_intensity = 0

remove_precursor_peak = 0

remove_precursor_tolerance = 1.5

clear_mz_range = 0.0 0.0

add_Cterm_peptide = 0.0

add_Nterm_peptide = 0.0

add_Cterm_protein = 0.0

add_Nterm_protein = 0.0

add_G_glycine = 0.0000

add_A_alanine = 0.0000

add_S_serine = 0.0000

add_P_proline = 0.0000

add_V_valine = 0.0000

add_T_threonine = 0.0000

add_C_cysteine = 57.021464

add_L_leucine = 0.0000

add_I_isoleucine = 0.0000

add_N_asparagine = 0.0000

add_D_aspartic_acid = 0.0000

add_Q_glutamine = 0.0000

add_K_lysine = 0.0000

add_E_glutamic_acid = 0.0000

add_M_methionine = 0.0000

add_O_ornithine = 0.0000

add_H_histidine = 0.0000

add_F_phenylalanine = 0.0000

add_U_selenocysteine = 0.0000

add_R_arginine = 0.0000

add_Y_tyrosine = 0.0000

add_W_tryptophan = 0.0000

add_B_user_amino_acid = 0.0000

add_J_user_amino_acid = 0.0000

add_X_user_amino_acid = 0.0000

add_Z_user_amino_acid = 0.0000

#

# COMET_ENZYME_INFO _must_ be at the end of this parameters file

#

[COMET_ENZYME_INFO]

0. No_enzyme 0 - -

1. Trypsin 1 KR P

2. Trypsin/P 1 KR -

3. Lys_C 1 K P

4. Lys_N 0 K -

5. Arg_C 1 R P

6. Asp_N 0 D -

7. CNBr 1 M -

8. Glu_C 1 DE P

9. PepsinA 1 FL P

10. Chymotrypsin 1 FWYL P

11. TrypN 0 KR -

12. NoEnzyme 0 J ABCDEFGHIJKLMNOPQRSTUVWXYZ

13. Tryp+Chymo 1 FKLRWY P

14. Tryp/P+Chymo/P 1 FKLRWY -
